# Laboratory information management system for COVID-19 non-clinical efficacy trial data

**DOI:** 10.1186/s42826-022-00127-2

**Published:** 2022-06-28

**Authors:** Suhyeon Yoon, Hyuna Noh, Heejin Jin, Sungyoung Lee, Soyul Han, Sung-Hee Kim, Jiseon Kim, Jung Seon Seo, Jeong Jin Kim, In Ho Park, Jooyeon Oh, Joon-Yong Bae, Gee Eun Lee, Sun-Je Woo, Sun-Min Seo, Na-Won Kim, Youn Woo Lee, Hui Jeong Jang, Seung-Min Hong, Se-Hee An, Kwang-Soo Lyoo, Minjoo Yeom, Hanbyeul Lee, Bud Jung, Sun-Woo Yoon, Jung-Ah Kang, Sang-Hyuk Seok, Yu Jin Lee, Seo Yeon Kim, Young Been Kim, Ji-Yeon Hwang, Dain On, Soo-Yeon Lim, Sol Pin Kim, Ji Yun Jang, Ho Lee, Kyoungmi Kim, Hyo-Jung Lee, Hong Bin Kim, Jun Won Park, Dae Gwin Jeong, Daesub Song, Kang-Seuk Choi, Ho-Young Lee, Yang-Kyu Choi, Jung-ah Choi, Manki Song, Man-Seong Park, Jun-Young Seo, Ki Taek Nam, Jeon-Soo Shin, Sungho Won, Jun-Won Yun, Je Kyung Seong

**Affiliations:** 1 Korea Mouse Phenotyping Center, Seoul National University, Seoul, 08826 Republic of Korea; 2 Institute of Health and Environment, Seoul National University, Seoul, 08826 Republic of Korea; 3 Department of Genomic Medicine, Seoul National University Hospital, Seoul, 03080 Republic of Korea; 4 Department of Medicine, Seoul National University College of Medicine, Seoul, 03080 Republic of Korea; 5 RexSoft Corp, Seoul, 08826 Republic of Korea; 6 Severance Biomedical Science Institute, Brain Korea 21 PLUS Project for Medical Science, Yonsei University College of Medicine, Seoul, 03722 Republic of Korea; 7 Institute of Immunology and Immunological Diseases, Yonsei University College of Medicine, Seoul, 03722 Republic of Korea; 8 Department of Microbiology, Yonsei University College of Medicine, Seoul, 03722 Republic of Korea; 9 Department of Microbiology, Institute for Viral Diseases, Biosafety Center, Korea University College of Medicine, Seoul, 02841 Republic of Korea; 10 Science Unit, International Vaccine Institute, Seoul, 08826 Republic of Korea; 11 Department of Laboratory Animal Medicine, College of Veterinary Medicine, Konkuk University, Seoul, 05029 Republic of Korea; 12 Department of Nuclear Medicine, Seoul National University Bundang Hospital, Seongnam, 13488 Republic of Korea; 13 Laboratory of Avian Diseases, BK21 Plus Program for Veterinary Science and Research Institute for Veterinary Science, College of Veterinary Medicine, Seoul National University, Seoul, 08826 Republic of Korea; 14 Korea Zoonosis Research Institute, Chonbuk National University, Iksan, 54531 Republic of Korea; 15 Department of Pharmacy, College of Pharmacy, Korea University, Sejong, 30019 Republic of Korea; 16 Bionanotechnology Research Center, Korea Research Institute of Bioscience and Biotechnology, Daejeon, 34141 Republic of Korea; 17 Division of Biomedical Convergence, College of Biomedical Science, Kangwon National University, ChunCheon, 24341 Republic of Korea; 18 Preclinical Research Center, Seoul National University Bundang Hospital, Seongnam, 13488 Republic of Korea; 19 Laboratory of Developmental Biology and Genomics, Research Institute for Veterinary Science, BK21 PLUS Program for Creative Veterinary Science Research, College of Veterinary Medicine, Seoul National University, Seoul, 08826 Republic of Korea; 20 Graduate School of Cancer Science and Policy, National Cancer Center, Goyang, Gyeonggi 10408 Republic of Korea; 21 College of Pharmacy, Dongguk University, Seoul, 04620 Republic of Korea; 22 Department of Biomedical Sciences and Department of Physiology, Korea University College of Medicine, Seoul, 02841 Republic of Korea; 23 Department of Periodontology, Section of Dentistry, Seoul National University Bundang Hospital, Seongnam, 13620 Republic of Korea; 24 Department of Internal Medicine, Seoul National University Bundang Hospital, Seoul National University College of Medicine, Seongnam, 13620 Republic of Korea; 25 Department of Public Health Sciences, Seoul National University, Seoul, 08826 Republic of Korea; 26 Laboratory of Veterinary Toxicology, College of Veterinary Medicine, Seoul National University, Seoul, 08826 Republic of Korea; 27 Interdisciplinary Program for Bioinformatics, Program for Cancer Biology, BIO-MAX/N-Bio Institute, Seoul National University, Seoul, 08826 Republic of Korea

**Keywords:** SARS-CoV-2, COVID-19, Non-clinical, Laboratory information management system, Data

## Abstract

**Background:**

As the number of large-scale studies involving multiple organizations producing data has steadily increased, an integrated system for a common interoperable format is needed. In response to the coronavirus disease 2019 (COVID-19) pandemic, a number of global efforts are underway to develop vaccines and therapeutics. We are therefore observing an explosion in the proliferation of COVID-19 data, and interoperability is highly requested in multiple institutions participating simultaneously in COVID-19 pandemic research.

**Results:**

In this study, a laboratory information management system (LIMS) approach has been adopted to systemically manage various COVID-19 non-clinical trial data, including mortality, clinical signs, body weight, body temperature, organ weights, viral titer (viral replication and viral RNA), and multiorgan histopathology, from multiple institutions based on a web interface. The main aim of the implemented system is to integrate, standardize, and organize data collected from laboratories in multiple institutes for COVID-19 non-clinical efficacy testings. Six animal biosafety level 3 institutions proved the feasibility of our system. Substantial benefits were shown by maximizing collaborative high-quality non-clinical research.

**Conclusions:**

This LIMS platform can be used for future outbreaks, leading to accelerated medical product development through the systematic management of extensive data from non-clinical animal studies.

## Background

The novel severe acute respiratory syndrome coronavirus 2 (SARS-CoV-2) has resulted in the serious respiratory illness pandemic, coronavirus disease 2019 (COVID-19). It was first reported in Wuhan, the capital of Hubei, China, in late December 2019 and has attracted international attention [1]. After four months, it spread to multiple countries, leading to more than one million confirmed cases worldwide [2]. SARS-CoV-2, a single-stranded RNA-enveloped virus [3], enters the host cells by binding its structural spike (S) protein to cell surface receptors, angiotensin-converting enzyme 2 (ACE2) [4]. After entering the host cells, the virus hijacks the cell to undergo viral replication [5]. During this process, an aberrant host immune condition called a cytokine storm can occur in response to SARS-CoV-2 infection, which is characterized by high concentrations of pro-inflammatory cytokines and chemokines, including tumor necrosis factor-α, interleukin-1, and interleukin-6 [6], resulting in severe COVID-19 associated with excessive inflammation [7].

Along with understanding the mechanism of transmissibility and pathogenesis of SARS-CoV-2, significant efforts are being devoted to developing novel vaccines and therapeutic agents to prevent or treat COVID-19. In particular, various COVID-19 related research has been conducted with the participation of multiple organizations. Among such studies, animal models, such as of monkeys, cats, ferrets, hamsters, and human angiotensin converting enzyme 2 (hACE2) transgenic mice [8], capable of conducting controlled experiments, have made significant contributions [9–13]. Because various parameters such as mortality, clinical signs, body weight, organ weights, viral titer (viral replication and viral RNA), and multiorgan histopathology should be comprehensively analyzed for a reliable interpretation of the results, there is an increasing need for an integrated system in which multiple institutions can apply a streamlined analysis and input the results simultaneously when conducting large-scale non-clinical efficacy tests of a COVID-19. When experimental data are collected from multiple institutions, “organization-level effects” may occur owing to non-standardization in the data coding process and coding errors, which can act as a batch effect in a data analysis. In large-scale experimental multicenter research, it is necessary to prevent the loss of reliability in the data analysis caused by batch effects; however, this is a difficult goal to achieve without an integrated system. An effective approach to overcoming the limitations caused by the batch effects mentioned above is to use a laboratory information management system (LIMS) [14]. LIMS systematizes the research process by automating data-related tasks that were conducted manually using information technology, and simultaneously realizes various needs, such as the standardization of experimental data, sharing, problem tracking, and the creation of reports based on document templates. The accumulated data stored in LIMS can be useful in understanding the current clinical knowledge and making decisions, which is a remarkable advantage in research on rapidly changing epidemics such as COVID-19. Despite the advantages of LIMS, no systems allowing multiple institutions to participate simultaneously in research related to COVID-19 have yet been proposed among the published LIMSs. Therefore, we aimed to clearly understand the standard procedures of non-clinical trials for COVID-19 therapeutics and vaccines and to design and implement an LIMS suitable for the research procedure.

## Results

### User management and functionalities

**Fig. 1:**
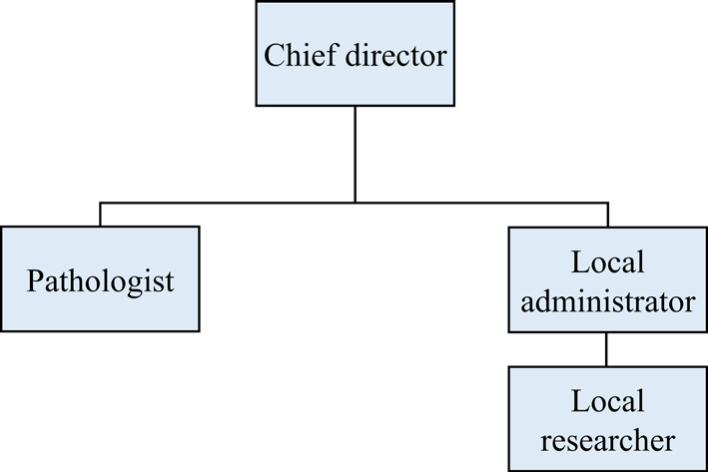
Authority organization chart. The proposed LIMS fully supports co-operated, multi-center study. In this respect, each institute can have pathologists and multiple local researchers and administrators, directed by a chief director

The COVID-19 non-clinical trial LIMS has four different types of users with different authorizations to handle multiple organizations: a chief director, a local administrator and researcher from each organization, and a pathologist (Fig. 1). The role of the chief director includes new project initiation, modification, deactivation, user account management, and data management. It can also control the registration of new organizations and the removal of existing organizations. The role of the local administrators is the same as that of the chief director, except that they are limited to the organization to which they belong. This enables the efficient management of each organization as well as data security. Researchers can manage the data entry/removal and check the project progress. Pathologists can conduct pathological analyses, pathological data entry, and related functions. Furthermore, multiple user types for multiple roles are allowed. If multiple authorizations are granted, the screen manual is shown based on the user’s choice.

The proposed system, which applies a convenient user interface, was created to provide many functions related to data management for multicenter research. The detailed function list is presented in Table 1. Functions can be classified into four different categories: access management, trial, audit, and comparison. The access management function forms the basis of the system and manages the user group. The trial function controls the entire project process, including initiation, progression, and finalization. The audit function records all activities that occur in the system and implements data entry, cancelation, and restoration. The comparison function provides several plots according to the investigational product (new drug/repositioning, vaccine/therapeutic), sponsor, experimental animals (hamster model/hACE2 mouse model), research objective, and study site. It performs access control and visualization by classifying all data accumulated in the system.

**Table 1:** Detailed function list of the proposed system

Category	Function	Note
Access & management	^+^ Management (C/U/D)	
	^※^ Join member	
	^※+*^ Grant member	
	Sign-in/out	
	Confirm EULA	Appeared once
	Manuals (system, injection demo, autopsy protocol/checklist/demo)	
Trial	^※+^ Initialize trial	
	^※*^ Activate trial	
	Participation confirmation	Appeared once
	^U^ Data management (run trial)	
	Visualize current data	
	^+*^ Export trial data	
	^AD^Additional dataset management	
	^※*^ Request pathological analysis	
	^†^ Perform pathological analysis	
	^※†^ Finish pathological analysis	
	^*^ Rollout trial report	
	^※*^ Request trial completion	
	^※+^ Complete trial	
Audit	Activity logging	Automatic
	^+^ Activity review and search (by trial, date, sample, or member)	
	^+^ Revert or recover the data management activity	
Comparison	Select trial (by type, objective, institution, model, data, institution)	
	Visualize the selected trials (by type, objective, model, institution)	

^C^Creation, ^U^Update, ^A^Activation, ^D^Deactivation

^+^Administrator-only function

^*^Principal investigator-only function

^†^Pathologist-only function

^※^Notified by e-mail or social messenger upon action

### Data security and audit

The proposed system provides several functions for data security and audits. For security, an access management system based on the access control list (ACL) [15] was incorporated to protect the data entry. The unintended data leakage was minimized by providing limited access points to users according to the user access levels. For each running instance, data security at the terminal level was obtained through secure HTTPS-based communication. For audits, the proposed system records, monitors, and manages all user activities, including data entry, by generating a separate database. Furthermore, it provides a history-tracking function to maintain data integrity. The chief director can identify the path of a data contamination and restore it to its original state, which minimizes the problem related to data entry or damage to the raw data.

### Process management

**Fig. 2:**
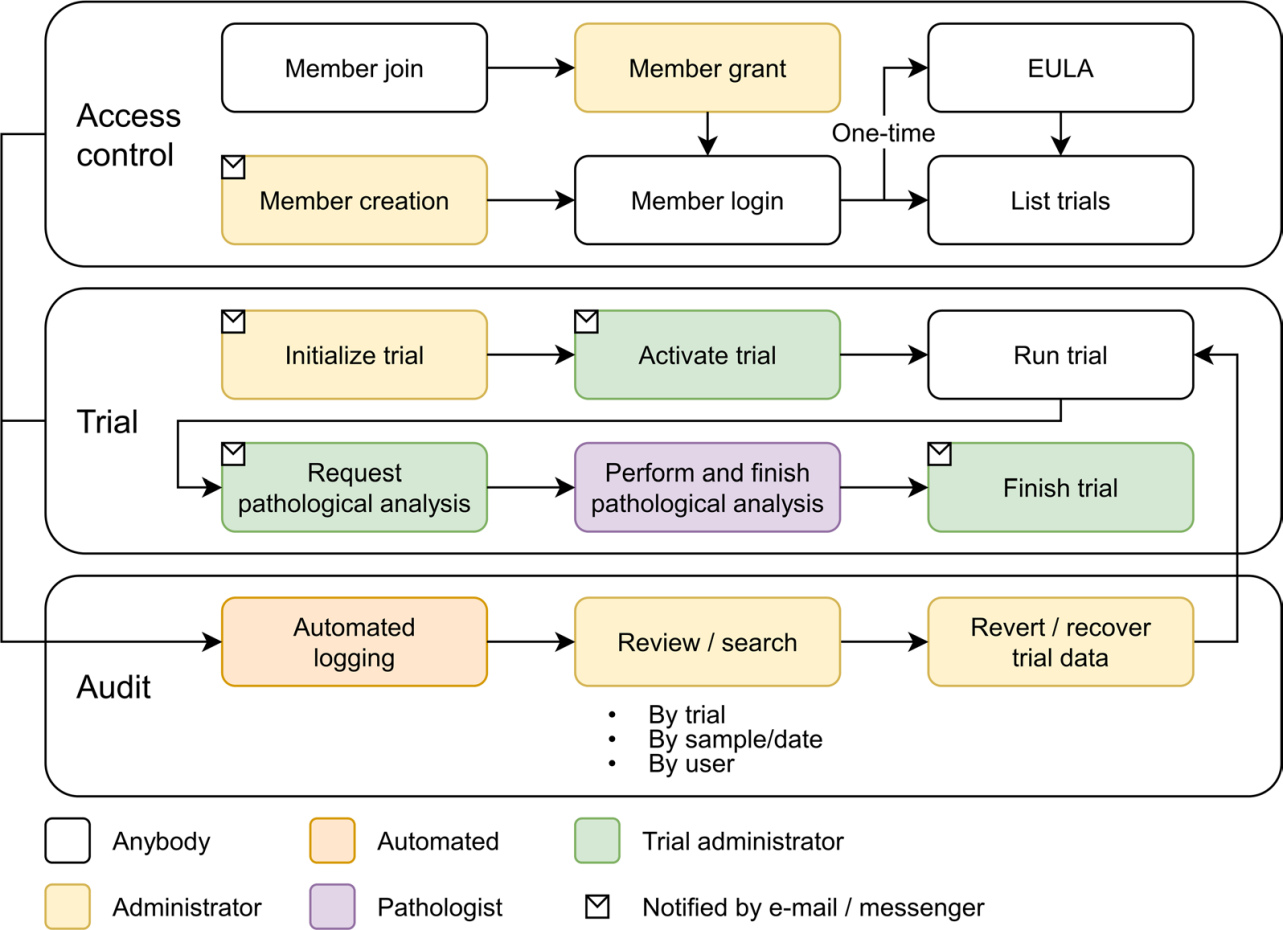
Recording workflow in LIMS. The proposed LIMS audits the activities and controls the access throughout the study. All the accesses to the trials are systematically consented and controlled with complete auditing. Therefore, the trial data can be tracked and managed using the audit record

Data entry and monitoring are possible for each institution, which makes it possible for the local administrator of each institution to check the data status in real time. It is also composed of a system in which the chief director supervises the overall project progress of each organization in real time. In addition, by systematically managing various types of non-clinical data and enabling collaboration between multiple organizations, researchers are expected to be able to quickly derive the desired results. Figure 2 shows the workflow of the COVID-19 non-clinical trial LIMS for vaccine and therapeutics treatments.

### Graphics and analysis functions

The COVID-19 non-clinical trial LIMS provides various visualizations that can review the data characteristics at each stage for each institution, as well as optimized visualizations such as line diagrams, bar diagrams, and survival curves according to the data type for awareness of current situations and early detection of unusual situations. The proposed system generates suitable plots for a correspondence analysis according to the data types. In particular, a comparison function was developed to facilitate mutual comparisons according to user-selected conditions, and the system can also generate easy-to-read study-specific graphs.

### Data downloading and result reporting

After obtaining approval from the chief director and administrators of each organization, the stored data can be downloaded as an Excel or Word file. In particular, it is possible to create an integrated Word file that includes a data description table, summary plot, and raw data, and all currently entered data can be downloaded as a multi-sheet Excel file with a single click for convenient data management. In addition, the proposed system can be used to monitor the data entry status of each participating organization and simultaneously create reports with a function for automatically summarizing the analysis results.

### Notification

In applying important commands such as research initiation and termination, a notification system through a mail and messenger (limited to South Korea) notification service was established for relevant individuals such as chief directors and administrators. When a major change occurs in the research, the manager can quickly identify and respond to any unintended situations.

### Data management and recording

**Fig. 3:**
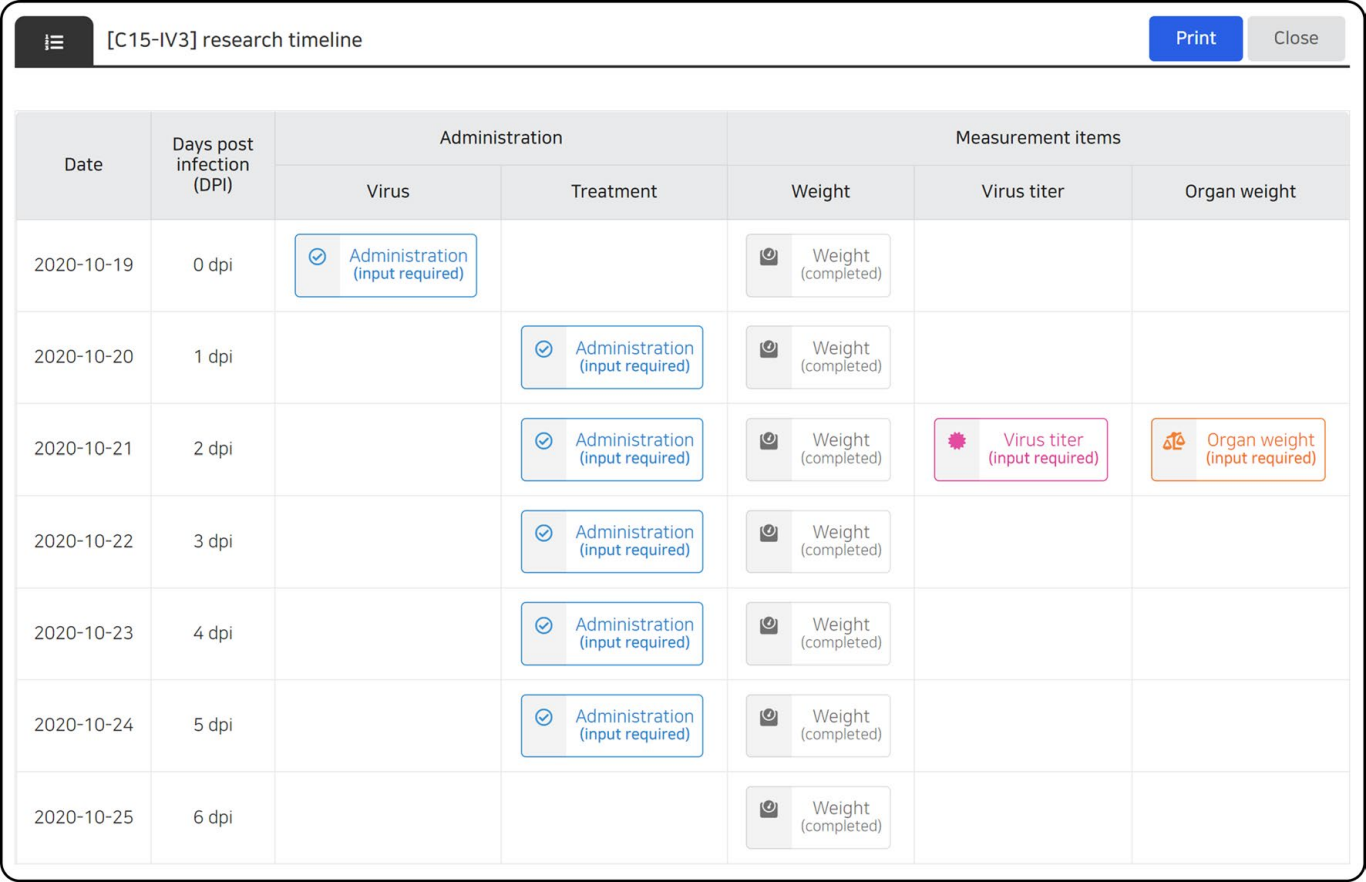
LIMS calendar. The proposed LIMS provides a calendar to facilitate research under the pre-deteremined study plan, which allows the researchers to review the overall schedule, and its corresponding data type and completion status at once

**Fig. 4:**
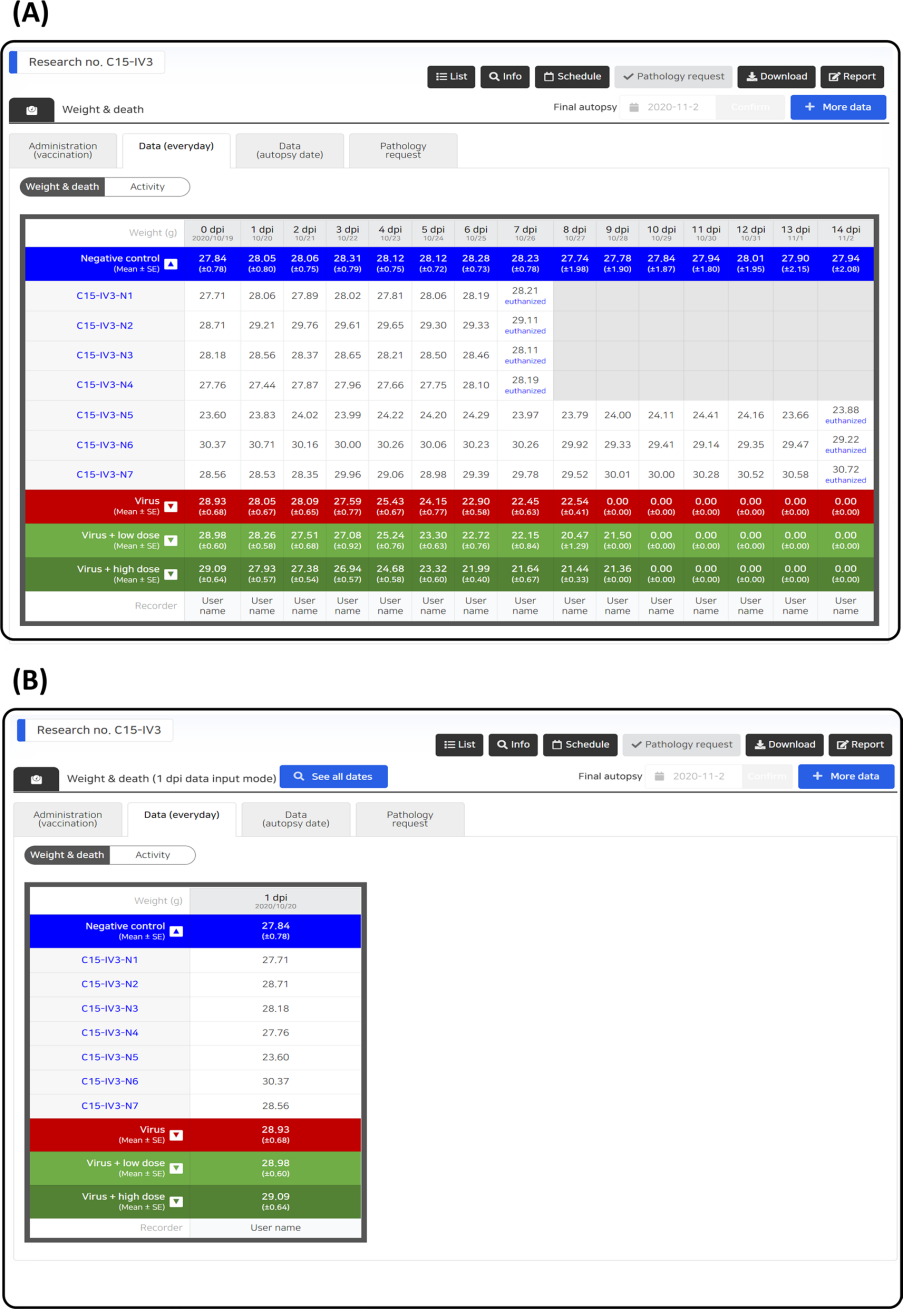
LIMS sample input. **A** The data entry screen for the entire schedule. **B** The data entry screen for the selected schedule

Data entry control can be achieved according to the characteristics of each data, and the user input is automatically controlled according to the condition of each stage of the study. Human error is minimized by limiting the range of unobservable values ​​for each data parameters, and entry omission is prevented by notifying the data that need to be input according to the investigation schedule, current date, and data entry status (Fig. 3). In addition, all data entry activities were recorded, and an audit function was reviewed for each dataset. If necessary, an unsuitable data entry can be canceled to prevent data errors. On the data entry screen, users can check all data during the full research period (Fig. 4A) or partial data by dividing the data based on date (Fig. 4B).

## Discussion

Non-clinical studies are essential for identifying the potential effects of promising drug candidates prior to entering human clinical trials [16]. Numerous animal studies have been conducted in the development of therapeutic agents and vaccines during the recent COVID-19 pandemic, leading to the need for rapid and accurate non-clinical efficacy analyses in standardized animal study design protocols [9, 10]. The inoculation of hACE2 transgenic mice with SARS-CoV-2 induced weight loss, increased the viral titer in the lungs, and histopathological changes with increased cytokine levels and interstitial pneumonia [17, 18]. In addition, infection with SARS-CoV-2 in a golden Syrian hamster model induced signs resembling COVID-19 disease, including decreased activity, weight loss, high lung viral load, and the presence of T-lymphocytes in the respiratory tract [19–21]. Therefore, systematic management of these extensive data, including mortality, clinical signs, body weight, body temperature, viral titer (viral replication and viral RNA), organ weights, and multiorgan histopathology, that can be observed in COVID-19 research using animal models such as hACE2 transgenic mice and hamsters is required for a reliable evaluation and development of a therapeutic agent or vaccine.

The implemented LIMS has many distinctive features providing many user-friendly functions. First, the implemented system classifies COVID-19 non-clinical trial topics into six sub-themes (investigational product, types of investigational product, sponsor, experimental animals, research objective, and study site). It also applies access control and visualization by classifying the research accumulated in the system. Second, using a streamlined analysis, it creates a four-step process related to the research progress, pathology analysis, entry of the results, and report generation. Access control and customized user interfaces are provided for each step, which highlights the work to be done and provides step-by-step guidance as a project planner. Third, a pathology analysis was incorporated to organize various types of pathology data and support the resulting input from pathologists. Finally, the data are organized systematically, and raw data, summary tables, and graph visualizations are documented. In addition, Table 2 provides brief summaries of other existing LIMS versions and the proposed COVID-19 non-clinical trial LIMS.

**Table 2:** Comparison with open- source LIMSs

	Bika LIMS	MendelLIMS	MetaLIMS	COVID-19
Data type	Not specific	Clinical samples	Environmental samples	Animal samples
Web-based/stand-alone	Web-based	Web-based	Web-based	Web-based
Implementation Software	Python	Javascript, Ruby	PHP	PHP, nodeJS
Database	ZODB	MySQL, PostgreSQL, SQLLite	MySQL	MySQL,
Website	https://github.com/bikalabs/bika.lims	http://dna-discovery.stanford.edu/software/mendelims/	https://github.com/cheinle/MetaLIMS/wiki	https://github.com/rexsoft-org/covid19-lims
COVID-19 optimized	No	No	No	Yes
Pathology report	Limited (customizable)	Limited (text only)	No	Yes (text + image)
Streamlined, multi-center study (customizable)	Limited	No	No	Yes
Reporting	Certificate PDF (Not COVID-19 specific, manual process is required)	Not supported	Not supported	COVID-19-specific, fully automated, Microsoft Word format

The COVID-19 non-clinical trial data management system developed in this study consists of a mode for the chief director, administrators, researchers of each organization, and pathologists. It aims to improve the completeness of the database by managing the data entry process for each institution. To confirm this, we applied the proposed system to an actual COVID-19 non-clinical multicenter study. For the actual application through the proposed system, thirtytwo COVID-19 non-clinical efficacy studies were conducted by six animal biosafety level-3 institutions in South Korea from July 2020 to February 2021 through the research funding. During the implementation period, the project progress was monitored by the chief director in real time through the management of the data entry process for each organization. In addition, the administrators of each institution were able to check and manage the data entry status of the researchers in real time. Researchers were able to review the status of the data entry by outputting descriptive statistics and graphics, such as histograms or box plots. The administrator of the institution mainly used the function for automatically generating a report based on the data entry, which enabled the efficient management and monitoring of the study.

As research on infectious diseases steadily increases after the COVID-19 pandemic, such studies are usually conducted as joint research involving multiple centers [22–26]. Therefore, a system for efficiently managing multicenter laboratory data is required, and standardization is essential for integrating the units of different studies. The COVID-19 non-clinical trial LIMS proposed in this study for organizing laboratory sample management, analysis, and reporting of the results can be beneficial for researchers in large-scale studies where multiple organizations participate in the production of data. The proposed system enables experimental data management, data sharing, and data analysis through a web-based interface. It provides various functions focusing on practical usabilities such as the construction of management systems, data quality control processes, various graphics and analysis modules, result reporting functions, and data security and notification functions by systematically implementing access management, test execution, auditing, and comparisons. Large-scale data can therefore be managed more quickly and effectively. In particular, the proposed system is focused on multicenter laboratory data collection and the prevention of various problems owing to the non-standardization of the data coding processes at each institution. Furthermore, it was shown through a streamlined analysis that multicenter non-clinical trials can be systematically conducted.

The contribution of the COVID-19 non-clinical trial LIMS toward vaccines and therapeutics has focused on providing an environment helping health care professionals and researchers establish new hypotheses to solve various problems in research by investigating trends related to COVID-19 prevention and treatment, as well as the use of collected databases. Therefore, we anticipate the high adaptability of our system in overcoming COVID-19. Despite these desirable properties, further challenging issues remain to be resolved. Representatively, advanced statistical analysis function modules should be applied to the system. Because the proposed system does not support analysis modules such as descriptive statistics, data accumulated in the system must be downloaded and processed individually when a statistical analysis is required. To complement this, a comparison function module was developed, and the researchers can compare the data ​​of each study in a graph by selecting the components to be compared. The development of linking the accumulated data in LIMS with a cloud-based statistical analysis program will bring considerable benefits for researchers in the near future.

## Conclusions

As research related to COVID-19 will continue to expand and become more complex in the future, the systematic storage and structuring of research data is expected to become more important. In conclusion, the present study provided a new usage of an LIMS, enabling efficient and reliable documentation, management, and reporting of the results. This tool will help researchers test therapeutic agents and vaccines against COVID-19, and can be adapted to different research projects. In addition to COVID-19 research, this LIMS platform can also be used for future diseases driven by pathogens currently unknown to result in a pandemic [27], leading to an accelerated development of medical products through the systematic management of extensive data from non-clinical animal studies.

## Methods

### COVID-19 data management system description

The COVID-19-specific LIMS was developed to manage accumulated data for a large-scale multicenter study of actual non-clinical efficacy trials for COVID-19. It provides several distinctive user-friendly features that are not provided in other COVID-19 LIMS (see Table 2 for details).

The COVID-19 non-clinical LIMS provides a web-based interface, and its implementation requires both front- and back-end developments [28]. The back-end of the COVID-19 LIMS was developed to run on PHP 7.0, MySQL 5.2, and nodeJS 11. Its front-end supports HTML5 [29] and can be run on a modern web browser. The proposed system functionality was built with external libraries such as PHPOffice, headless chromium, and semantic UI. All components are accessible by request to the authors. The main web page for the proposed system is shown in Fig. 4.

## Data Availability

The datasets used and/or analyzed during the current study are available from the corresponding author on reasonable request.

## Abbreviations

ACE2: Angiotensin-converting enzyme 2
ACL: Access control list
COVID-19: Coronavirus disease 2019
LIMS: Laboratory information management system
SARS-CoV-2: Severe acute respiratory syndrome coronavirus 2
